# Photovoltaic cell fabricated using nanoporous black silicon synthesized *via* aluminium-assisted chemical etching†

**DOI:** 10.1039/d5ra01468a

**Published:** 2025-06-03

**Authors:** Shahnawaz Uddin, Nur Afidah Md Noor, Md Roslan Hashim, Mohd Zamir Pakhuruddin

**Affiliations:** a School of Physics, Universiti Sains Malaysia 11800 Minden Penang Malaysia shahnawazuddin.uwp@amu.ac.in; b University Women's Polytechnic, Aligarh Muslim University Aligarh 202002 India; c JA Solar Malaysia Sdn. Bhd. Lot 17001, Medan Bayan Lepas, Phase 4, Bayan Lepas Industrial Park 11900 Penang Malaysia; d Institute of Nano Optoelectronics Research and Technology (INOR), Universiti Sains Malaysia 11800 USM Penang Malaysia

## Abstract

In this work, a photovoltaic (PV) cell fabricated using nanoporous black silicon (bSi) synthesized *via* an aluminium-assisted chemical etching (AACE) process is demonstrated for the first time. To fabricate the PV cells, n-emitters are diffused into the p-type planar crystalline silicon (cSi) and nanoporous bSi substrates to form pn-junctions, followed by the deposition of metal contacts (*i.e.*, silver-grid as front contact and aluminium as back contact). The highest average power conversion efficiency (*η*_avg_) of 18.62% is obtained from the PV cell fabricated using the nanoporous bSi sample with the lowest average reflection of 5.7% within the 300–1100 nm wavelength region. This efficiency represents an enhancement of 169% when compared to the average efficiency of 6.93% for the reference planar cSi PV cell fabricated under similar conditions. The significant efficiency improvement is attributed to the superior broadband light trapping by the nanoporous bSi surface, which effectively suppresses optical reflection across a wide spectral range. The power conversion efficiency of the bSi PV cell will be further enhanced by incorporating an ARC and a passivation material on the device.

## Abstract 2

Nanoporous black silicon (bSi) synthesized *via* aluminium-assisted chemical etching process is being used in the fabrication of photovoltaic cells which produce a maximum average power conversion efficiency of 18.62% without any passivating material.

## Graphical abstract

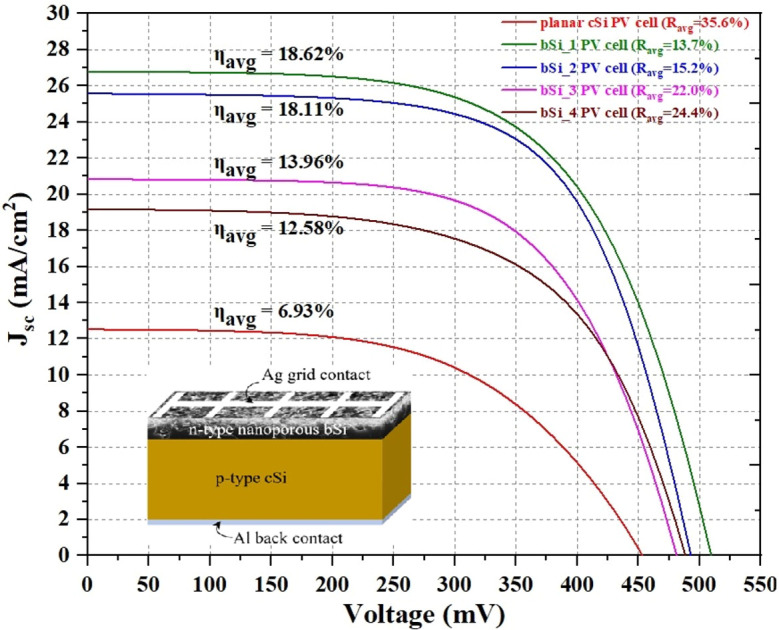

## Introduction

Crystalline silicon (cSi) PV cells currently dominate about 95% of the photovoltaic industry due to the abundance of silica in the earth's crust, high device efficiency, proven stability in the field, non-toxicity and reducing technology cost.^1,2^ In its original form, cSi exhibits a high broadband reflection within the 300–1100 nm spectral region, which is undesirable for PV conversion in the solar cells.^2,3^ As a potential solution, black silicon (bSi) offers superior broadband anti-reflection within the same wavelength range due to the presence of nanotextures on the surface of the cSi wafers, which induce refractive index grading effect.^4^ Owing to its superior broadband light absorption, bSi exhibits promising results for the photovoltaic cells even without incorporating a conventional anti-reflective coating (ARC) layer such as silicon nitride (SiN_*x*_) on the nanotextures.^5^ Furthermore, bSi demonstrates omni-directional light absorption capability which makes it less sensitive to illumination angle from the sun.^4,6^

Metal-assisted chemical etching (MACE) process is commonly employed to produce the nanotextured bSi since it is facile, quick, inexpensive and scalable.^7^ For the MACE process, several catalysts have been investigated including silver (Ag), gold (Au), platinum (Pt), copper (Cu) and nickel (Ni).^8–11^ Due to high resistance to oxidation, Au is commonly used as the catalyst in the MACE process, but as a deep-level trap, it decreases mobility of charge carriers, their lifetime and diffusion length especially in the minority carriers-based semiconductor devices such as PV cells. Therefore, catalysts other than Au are preferred for the fabrication of the bSi for PV cell applications.^12^ Very recently, aluminium (Al) has been employed by our group as an effective and economical catalyst to fabricate nanoporous bSi with a precise control over the surface-morphology *via* aluminium-assisted chemical etching (AACE) process.^13–16^ In addition, Al is abundant, environment friendly (*i.e.*, less toxic) and compatible with the manufacturing process of PV industry as being used as a metal contact of the PV cells.^17–19^

For the industrial production of high efficiency PV cells, MACE process is more suitable for texturing the surface of silicon. Generally, the optimally designed bSi based PV cells with or without antireflective coating exhibit a high-power conversion efficiency compared to the standard planar cSi based PV cells. A decent efficiency of 22.6% was demonstrated by PV cell based on the bSi produced by CuSO_4_ MACE process reported by Zhang *et al.*^20^ which is comparable to the industrial level solar panels (22.8%) developed by SunPower (a commercially manufacture of PV cells) in 2022.^21^ Some of the state of the art of PV cells based on bSi fabricated by MACE process using different metal catalysts have been listed in Table 1. Based on the table, it is evident that there is no previous work that has systematically investigated Al as the catalyst in the MACE process to fabricate nanoporous bSi for PV cell application.

**Table 1:** State-of-the-art PV cells based on bSi synthesized *via* MACE process using different metal catalysts

Base material/substrate	Metal catalyst	ARC/passivation	*J*_sc_ (mA cm^−2^)	*V*_oc_ (mV)	FF (%)	*η* (%)
p-Type mono cSi	Au	—/SiO_2_	35.6	615	78.2	17.1 (ref. 22)
p-Type mono cSi	Ag	—/SiO_2_	36.5	628	79.6	18.2 (ref. 23)
p-Type mono cSi	Ag	—/Al_2_O_3_	41.3	598	75.1	18.2 (ref. 24)
p-Type multi cSi	Ag	SiN_*x*_/—	36.0	634	79.2	18.0 (ref. 25)
p-Type mono cSi	Ag	SiN_*x*_/—	36.5	646	80.5	19.0 (ref. 25)
p-Type multi cSi	Ag	SiN_*x*_/—	36.7	634	79.3	18.5 (ref. 26)
p-Type mono cSi	Cu	—/SiO_2_	36.6	616	75.4	17.0 (ref. 27)
p-Type multi cSi	Ag/Cu	SiN_*x*_/—	36.8	632	80.2	18.7 (ref. 28)
p-Type multi cSi	Ag	SiN_*x*_/Al_2_O_3_	39.2	667	79.1	20.7 (ref. 29)
p-Type mono cSi	Ag	None	41.6	613	77.6	19.8 (ref. 30)
p-Type mono cSi	Cu	SiN_*x*_/—	37.5	638	78.8	18.9 (ref. 31)
p-Type multi cSi	Ag	SiN_*x*_/—	36.3	632	79.1	18.1 (ref. 32)
p-Type mono cSi	Ag	SiN_*x*_/—	38.5	647	80.9	20.2 (ref. 33)
p-Type multi cSi	Ag	SiN_*x*_/SiO_2_	38.4	641	78.4	19.3 (ref. 34)
p-Type multi cSi	Ag	SiN_*x*_/—	37.6	634	80.1	19.1 (ref. 35)
p-Type multi cSi	Ag	SiN_*x*_:H/—	36.9	637	80.3	18.9 (ref. 36)
p-Type mono cSi	Cu	SiN_*x*_/SiO_*x*_–Al_2_O_3_	41.2	680	80.6	22.6 (ref. 20)

In this work, PV cell based on nanoporous bSi fabricated *via* AACE process (with Al as the catalyst) is demonstrated for the first time. The as-synthesized samples of nanoporous bSi (the details of synthesis is reported in our earlier work^16^) along with the planar cSi (as a reference) are utilized to fabricate pn homojunction *via* phosphorus diffusion process at a temperature of 950 °C. The as-fabricated planar cSi and nanoporous bSi pn junctions are characterized (*i.e.*, morphological, optical and electrical characterizations). Finally, the front (silver) and back (aluminium) metal contacts are deposited on the pn junctions to complete the fabrication of PV cells. The nanoporous bSi PV cells are investigated and analyzed in comparison to a reference planar cSi PV cell fabricated under similar conditions. From the results, the highest average power conversion efficiency (*η*_avg_) of 18.62% has been achieved by the PV cell based on nanoporous bSi fabricated by the AACE process. In comparison, efficiency of 6.93% has been obtained for the reference planar cSi PV cell.

## Materials and methods

The nanoporous bSi samples are synthesized *via* AACE process as described in the ESI.† The as-fabricated nanoporous bSi samples along with the planar cSi substrate (for a reference) undergo a spin coating (at 3000 rpm for 20 s) to deposit a thin layer of the emulsion of 25% (v/v) phosphoric acid (H_3_PO_4_) dissolved in an organic solvent, 2-butanol (C_4_H_10_O).^37^ Then, the emulsion coated samples are baked on a hot-plate at 350 °C for 5 minutes to transform H_3_PO_4_ into a thin layer (∼1.1 μm) of oxide of phosphorus (*i.e.*, P_2_O_5_) *via* the process of evaporating C_4_H_10_O and decomposition of H_3_PO_4_ through the dehydration and condensation process as per eqn (1).^37,38^ It should be noted that 2-butanol has been preferred over the other organic solvents (such as methanol and ethanol) since it evaporates slowly due to its higher boiling temperature (117 °C) as compared to other organic solvents leading to a uniform-stable layer of P_2_O_5_. The uniform layer of P_2_O_5_ helps in the uniform vertical diffusion of phosphorus which results in a uniform sheet resistance over the whole surface of the samples.^38^ To get a minimum contact resistance with the n-emitter, the P_2_O_5_ coated samples are thermally annealed at 950 °C for 22 minutes.^38^ The annealing process converts deposited P_2_O_5_ on planar c-Si and nanoporous b-Si samples into phosphorus (P) through an interface reaction as per eqn (2) and a phosphorus-diffused n-emitter is generated under the set conditions.^37,38^1
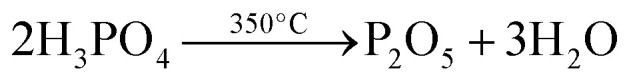
2
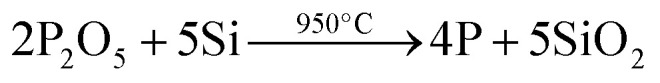


A layer of residual phosphosilicate glass, (P_2_O_5_)_*x*_ (SiO_2_)_1−*x*_, is also formed on the top-surface of the n-emitter during the diffusion process which is removed by dipping the samples in an aqueous solution of HF and HNO_3_ (5 ml of HF (48%) + 3.5 ml of HNO_3_ (70%) + 100 ml of H_2_O) for ∼3 minutes depending on the thickness of the PSG layer.^37^ Then, the samples are rinsed in H_2_O and dried by N_2_ gas. After the PSG removal process, the shunting problem of the pn-homojunction across the edges is removed by the edge-isolation *via* trimming the edges of the samples from all sides with a sharp diamond scriber.^39,40^

After fabricating the pn-homojunctions (*i.e.*, n-emitters on top surface of the p-type planar cSi and nanoporous bSi samples), the morphological, optical and electrical characterizations are done using field emission scanning electron microscope (FESEM, model: NOVASEM 450), atomic force microscope (AFM, model: Dimension Edge, Bruker), Agilent's UV-Vis-NIR spectrophotometer (model: Cary 5000) and Hall effect system (HL5500PC, model: LAKESHORE Controller 601DRC-93CA) respectively. Hall effect measurements are done using Van der Pauw geometry method at room temperature (25 °C), probe current of 0.129 mA and magnetic field strength of 0.520 tesla. During the optical characterization, the total reflection, *R*(*λ*) (diffused + specular) is measured with an incident angle (8°) of the light source. The ImageJ software tool is used to analyze the FESEM images for evaluating average depth, average diameter and surface coverage of the nanopores. AFM images are used to calculate the average root mean square (RMS) surface roughness. From the optical results, the weighted average reflections (*R*_avg_) of the planar cSi and nanoporous bSi samples are evaluated by using the following eqn (3), within 300–1100 nm wavelength range; where *S*(*λ*) is photon spectral density (PSD) under AM1.5 G (standard solar spectra).^13–16^3
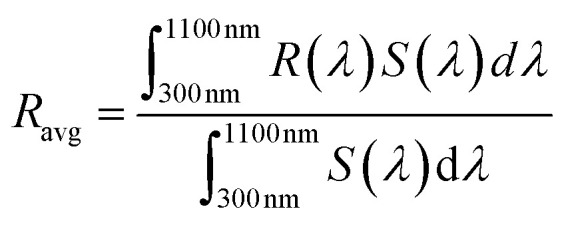


**Fig. 1:**
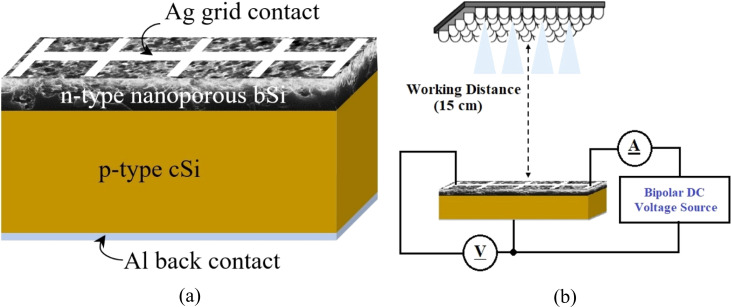
(a) Schematic diagram of nanoporous bSi PV cell (b) *J*–*V* measurement system.

The electrical parameters of the n-emitters measured by Hall effect system include sheet resistance (*R*_sheet_), electron mobility (*μ*_e_) and charge carrier concentration (*n*_e_).^41^ To complete the fabrication of PV cells, the pn-homojunctions undergo the metal contact deposition (Ag for front grid-contact and Al for back-contact) of around 300 nm thickness using a thermal evaporator (Edwards Auto 306). The metal contacts of pn-homojunctions are fired in a furnace at 600 °C for 22 minutes for the purpose of relieving stress by inducing the desirable reactions at the metal–semiconductor interface. The co-firing process of metal contacts at 600 °C also grows a thin SiO_2_ (<70 nm) acting as a self-passivation layer (an accrued advantage) on the surface of n-emitter (n-type nanoporous bSi) in the presence of residual oxygen in an N_2_ ambient.^42^ This passivating layer of SiO_2_ reduces the surface recombination to some extent.^43^ The PV cells fabricated are then characterized by LED solar simulator (model: TMS-2X2 Forter Technology) at an illumination power of 45 mW cm^−2^ at room temperature (25 °C), which measures short-circuit current density (*J*_sc_), open-circuit voltage (*V*_oc_), fill factor (FF) and power conversion efficiency (*η*).^41^ The schematic diagram of the nanoporous bSi PV cell along with current density–voltage (*J*–*V*) measurement system is illustrated in Fig. 1.

## Results and discussions

**Fig. 2:**
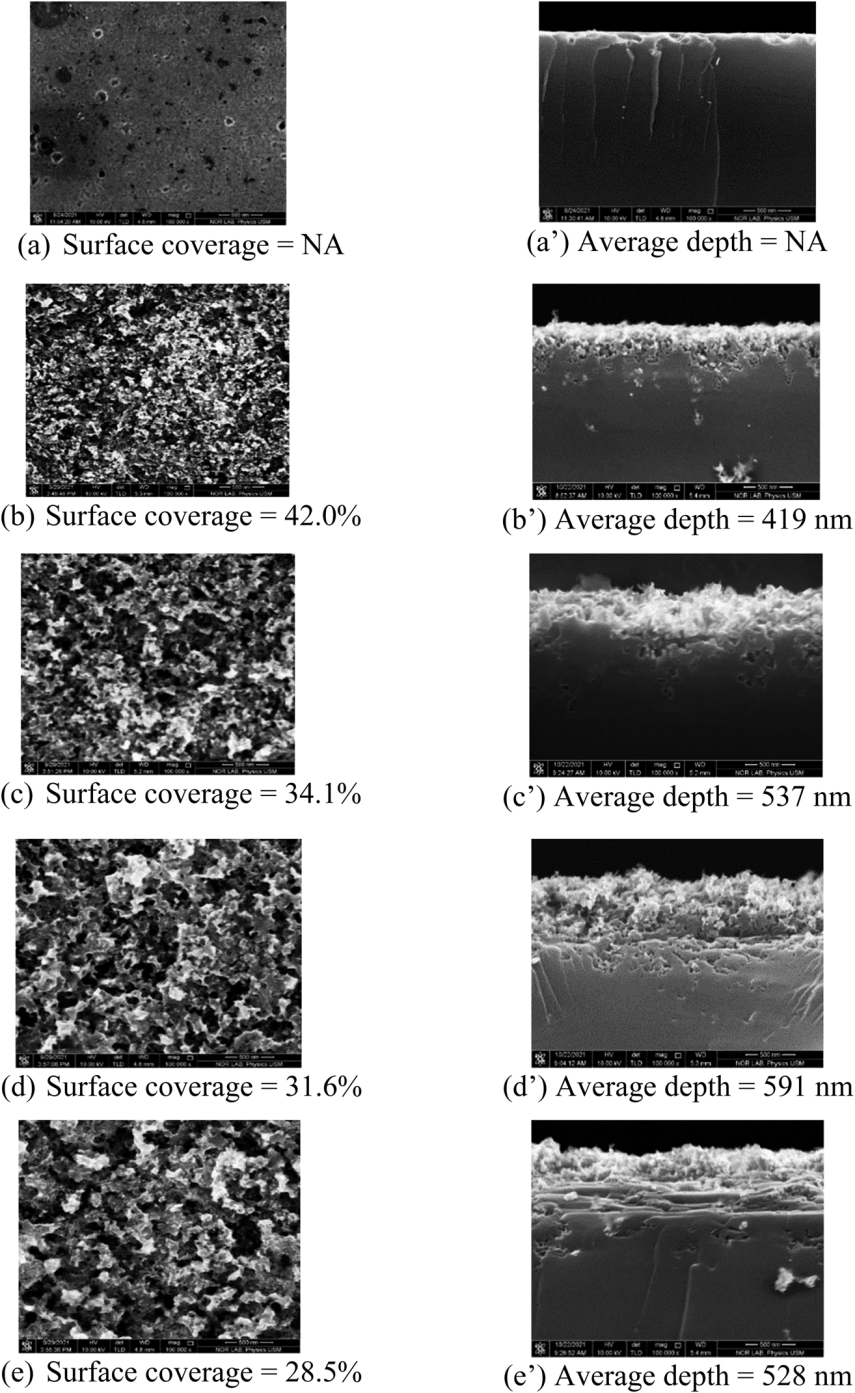
Top and cross-sectional FESEM images of pn-homojunctions as-fabricated on planar cSi and nanoporous bSi fabricated *via* AACE process with a varying chemical composition of HF–H_2_O_2_–H_2_O: (a and a′) Planar cSi, (b and b′) bSi_1 with HF–H_2_O_2_–H_2_O (10–1–10 ml), (c and c′) bSi_2 with HF–H_2_O_2_–H_2_O (10–4–10 ml), (d and d′) bSi_3 with HF–H_2_O_2_–H_2_O (10–7–10 ml), (e and e′) bSi_4 with HF–H_2_O_2_–H_2_O (10–10–10 ml).

Fig. 2 illustrates the top and cross-sectional FESEM images of the pn-homojunctions fabricated on the planar cSi and nanoporous bSi samples fabricated *via* the AACE process for the different chemical compositions of HF–H_2_O_2_–H_2_O (10–*x*–10 ml). The surface morphological parameters of the samples are summarized in Table 2. If compared with the morphology of the nanoporous bSi samples as listed in Table S1,† there is a slight modification in the top surface morphology after diffusion of n-emitter and PSG removal process. The average depth of the nanopores has been slightly reduced while the average diameter has been increased due to the etching in lateral direction only during the PSG removal process, since there is no Al catalyst on the surface after the n-emitter diffusion. The surface coverage by the nanopores has also been reduced because of elimination of shallow nanopores during the PSG removal.^13^ In addition to the surface morphology, the optical and electrical characteristics are also affected after PSG removal.^44^

**Table 2:** Surface morphological properties of top surface of the pn-homojunctions as-synthesized on the planar cSi and nanoporous bSi samples fabricated *via* the AACE process for a varying chemical composition of HF–H_2_O_2_–H_2_O

pn homojunction synthesized on samples	Average depth (nm)	Average diameter (nm)	Surface coverage (%)	RMS surface roughness (nm)
Planar cSi	NA	NA	NA	14.7
bSi_1	419	38.7	42.0	58.9
bSi_2	537	44.6	34.1	70.3
bSi_3	591	46.5	31.6	106.0
bSi_4	528	48.9	28.5	94.7

**Fig. 3:**
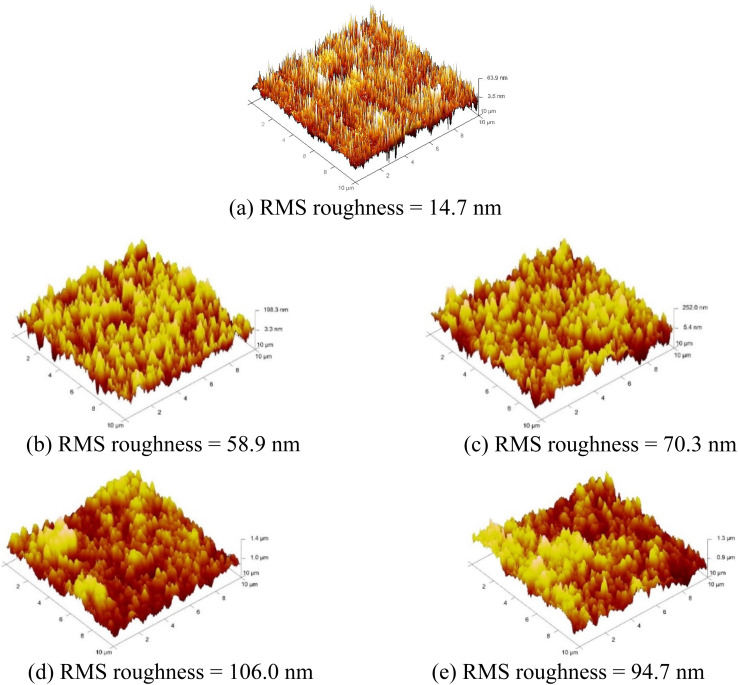
AFM images of pn junctions produced on planar cSi and nanoporous bSi fabricated *via* AACE process with a varying chemical composition of HF–H_2_O_2_–H_2_O: (a) planar cSi (b) bSi_1 with HF–H_2_O_2_–H_2_O (10–1–10 ml) (c) bSi_2 with HF–H_2_O_2_–H_2_O (10–4–10 ml) (d) bSi_3 with HF–H_2_O_2_–H_2_O (10–7–10 ml) (e) bSi_4 with HF–H_2_O_2_–H_2_O (10–10–10 ml).

AFM characterization measures the RMS surface roughness of the top surface of pn-junctions produced on planar cSi and nanoporous bSi fabricated *via* AACE process (see Fig. 3). As summarized in Table 2, the RMS roughness of pn-junction produced on planar cSi sample is 14.7 nm. The RMS roughness of the top surface of pn-junctions produced on nanoporous bSi increases for higher volume concentrations of H_2_O_2_ (1–7 ml) due to rapid and increased etching of silicon in the presence of high availability of h^*+*^. Further increase in H_2_O_2_ concentration (10 ml) causes a slight reduction in the RMS surface roughness due to excess diffusion of h^*+*^ in all directions even to the top ends of the nanopores which are trimmed by the etching process and result in lower RMS roughness. In other words, we can say that RMS surface roughness follows the trend of the average depth of the nanopores.

**Fig. 4:**
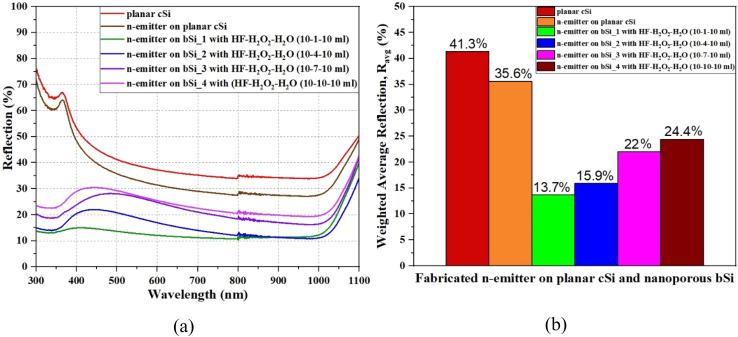
(a) The reflection profiles and (b) the weighted average reflection (*R*_avg_); from the n-emitters as-fabricated on the planar cSi and nanoporous bSi substrates.

**Fig. 5:**
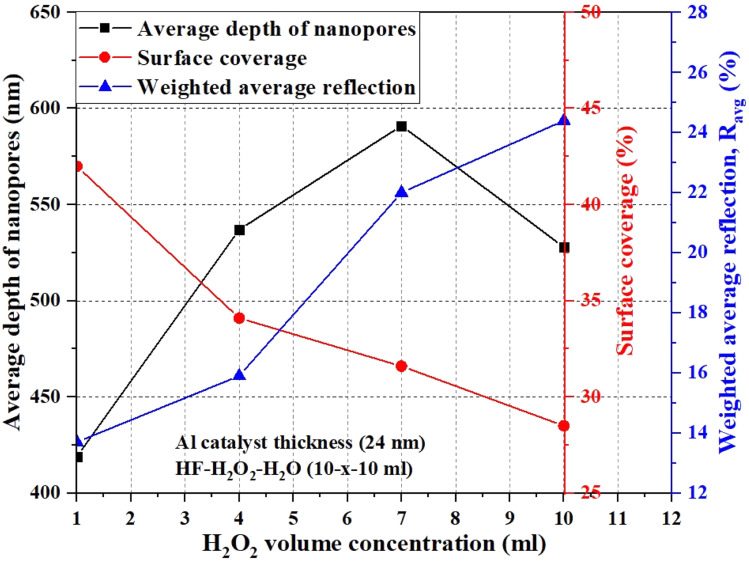
Correlation of the average depth, surface coverage and R_avg_ of pn-junctions produced on nanoporous bSi fabricated with a varying chemical composition of HF–H_2_O_2_–H_2_O (10–*x*–10 ml) by varying the volume concentration of H_2_O_2_ (1–10 ml).

The broadband light reflection curves (within 300–1100 nm wavelength range) of the as-synthesized n-emitters on the planar cSi and nanoporous bSi substrates are illustrated in Fig. 4. A small step change in the light reflection can be observed in Fig. 4(a) because of switching of integrated light sources in the spectrophotometer at a wavelength of 800 nm during the measurement of specular/diffused reflection.^13–16^ The high average broadband reflection (∼40%) in planar cSi is attributed to a step change in refractive index from air to cSi (*i.e.*, for air, *n*_air_ = 1 and for cSi, *n*_cSi_ = 4) while graded refractive index effect in nanopores increases the incident light trapping which in turn reduces the broadband light reflection.^13–16^ The broadband reflection as well as *R*_avg_ from the surface of n-emitter on the planar cSi and nanoporous bSi substrates is lower as compared to that from the planar cSi/nanoporous bSi substrates because of the increased RMS roughness (Tables 2 and S1†). As shown in Table 3, in case of n-emitter on planar cSi sample, *R*_*avg*_ has been reduced from 41.3% to 35.6% owing to higher value of RMS roughness. While in case of n-emitters fabricated on the nanoporous bSi samples, the *R*_avg_ (13.7%, 15.9%, 22.0% and 24.4%) has been increased in comparison to the respective values of *R*_avg_ (5.7%, 8.9%, 12.7% and 14.3%) obtained from the same samples of nanoporous bSi as reported in our earlier work.^16^ Herein, an increase in *R*_avg_ is due to the altered morphology (*i.e.*, average depth/diameter and surface coverage), especially a reduction in the average depth and the surface coverage of nanopores because of PSG removal after diffusion of n-emitter and a reduced porosity (an increase in refractive-index) of nanoporous bSi by high temperature treatment (>572 °C) during diffusion process at 950 °C.^45,46^ The correlation of average depth of nanopores, surface coverage and *R*_avg_ of n-emitter fabricated on nanoporous bSi samples synthesized *via* AACE process with a varying volume concentration of H_2_O_2_ is illustrated in Fig. 5 which explains that *R*_avg_ is minimum when the shallower and denser nanopores are synthesized for a lower volume concentration of H_2_O_2_ (1 ml).

**Table 3:** *R*_avg_ of the planar cSi and nanoporous bSi samples fabricated with a varying chemical composition of HF–H_2_O_2_–H_2_O (10–*x*–10 ml) (before/after diffusing n-emitter)

Sample name	Chemical composition, HF–H_2_O_2_–H_2_O (v–v–v) ml–ml–ml	Weighted average reflection, *R*_avg_ (%)	
		Before n-emitter	After n-emitter
Planar cSi	—	41.3	35.6
bSi_1	10–1–10	5.7	13.7
bSi_2	10–4–10	8.9	15.9
bSi_3	10–7–10	12.7	22.0
bSi_4	10–10–10	14.3	24.4

Hall effect results of the n-emitter formed after the diffusion process are presented in Table 4. From these results, it is observed that all the n-emitters diffused into either planar cSi or nanoporous bSi are moderately doped (*i.e.*, *n*_e_ = 1.62 × 10^17^ cm^−3^ to 5.67 × 10^17^ cm^−3^). The sheet resistance (*R*_sheet_) of the n-emitter diffused into the planar cSi is 24.42 Ω sq^−1^ while for the n-emitter diffused into the nanoporous bSi samples, it is in the range of 11.68 Ω sq^−1^ to 18.36 Ω sq^−1^. Owing to the porous nature of top surface of bSi, the diffusion of phosphorus in nanoporous bSi is faster and deeper as compared to the planar cSi. Thus, the n-emitters diffused into the nanoporous bSi exhibit lower sheet resistances which are also confirmed by Oh *et al.* (55 Ω sq^−1^), Stilling-Andersen *et al.* (74 Ω sq^−1^) and Li *et al.* (50 Ω sq^−1^).^23,47,48^ Correlating the RMS roughness of nanoporous bSi samples from Table 2 and the sheet resistance in Table 4, the sheet resistance of the n-emitter fabricated on the samples (bSi_1 to bSi_4) increases with the RMS roughness which may be attributed to the shallow depth of n-emitters in case of nanoporous bSi sample with a higher RMS roughness. To produce good ohmic contact, the n-emitter should be highly doped but higher doping concentration contributes to enhanced recombinations of charge carriers which results in lower values of *V*_oc_ and degrades the performance of the PV cells with time. Therefore, a compromise is made between a good ohmic contact and a lower recombination current produced by fabricating a moderately doped n-emitter.^49^ The electron mobility (*μ*_e_) in the n-emitter of planar cSi is 54.2 cm^2^ V^−1^ s^−1^ while the electron mobilities in the n-emitters of different samples of nanoporous bSi range from 33.7 cm^2^ V^−1^ s^−1^ to 46.7 cm^2^ V^−1^ s^−1^. The porosity and RMS surface roughness of the n-emitters diffused into nanoporous bSi affect the mobility of the charge carriers. Furthermore, an enhanced surface area of nanoporous bSi introduce defects or impurities, leading to impurity scattering which causes reduced mobility of charge carriers, increased surface recombinations and defect-induced bandgap states. This impurity scattering effect in bSi-based solar cells reduces efficiency by limiting carrier mobility, increasing recombination and lowering open circuit voltage. Managing these effects through surface passivation and precise fabrication is key to realizing the full potential of the bSi.

**Table 4:** The electrical parameters of n-emitter of pn junctions fabricated on planar cSi and nanoporous bSi as-measured by Hall effect system

pn homojunction as-fabricated on sample	Sheet resistance		Carrier mobility		Carrier concentration	
	Before n-emitter *R*_sheet_ (Ω sq^−1^)	After n-emitter *R*_sheet_ (Ω sq^−1^)	Before n-emitter, *μ*_h_ (cm^2^ V^−1^ s^−1^)	After n-emitter, *μ*_e_ (cm^2^ V^−1^ s^−1^)	Before n-emitter, *n*_h_ (cm^−3^)	After n-emitter, *n*_e_ (cm^−3^)
Planar c-Si	273.1	24.42	235	54.2	3.15 × 10^15^	1.62 × 10^17^
b-Si_1	279.6	11.68	147	33.7	4.64 × 10^15^	5.67 × 10^17^
b-Si_2	296.3	12.92	163	44.0	4.81 × 10^15^	3.93 × 10^17^
b-Si_3	302.0	18.36	170	46.7	3.33 × 10^15^	2.60 × 10^17^
b-Si_4	290.5	17.69	157	41.3	4.71 × 10^15^	3.05 × 10^17^

**Fig. 6:**
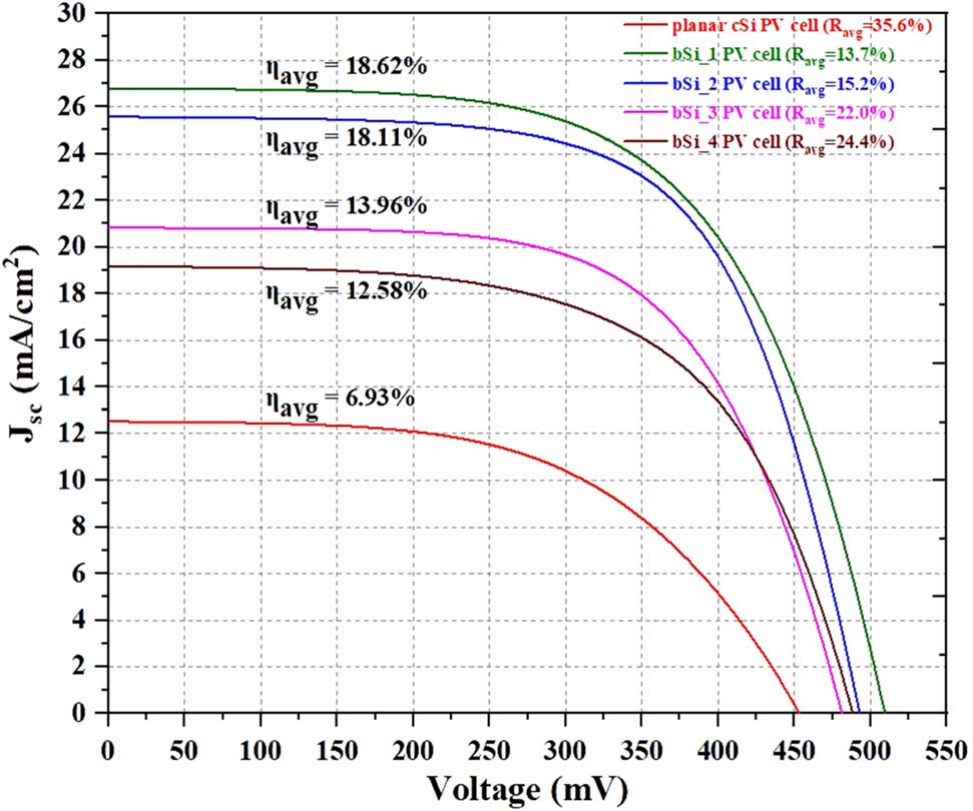
*J*–*V* characteristics of the planar cSi and nanoporous bSi PV cells. Planar cSi PV cell is used as a reference.

Table 5 summarizes the average values of electrical parameters of the planar cSi and nanoporous bSi PV cells. The planar cSi PV cell is used as a reference. The measurements are carried out using a white light LED based solar simulator with an input intensity (*P*_in_) of 45 mW cm^−2^ at the room temperature (*T* = ∼25 °C). The power conversion efficiency (PCE) for a PV cell is calculated by using the following formula, *η* = *(J*_sc_ × *V*_oc_ × FF*)/P*_in_.^50^ Since the white light LED source generates the light intensity within a narrow wavelength of 400–800 nm, relatively lower values of *J*_sc_ for the PV cells are expected as compared to that obtained under standard test condition (STC) (AM1.5G).^41,51,52^ The *J*–*V* curves for the as-fabricated PV cells by using different samples of the planar cSi and nanoporous bSi are shown in Fig. 6.

**Table 5:** The average values of the electrical parameters of the planar cSi and nanoporous bSi PV cells. The measurements are carried out under a white light LED solar simulator with an input intensity (*P*_in_) of 45 mW cm^−2^

PV cell fabricated on sample	*J*_sc_ (mA cm^−2^)	*V*_oc_ (mV)	FF (%)	*η*_avg_ (%)
Planar cSi	12.5	453.9	54.9	6.93
bSi_1	26.8	510.1	61.3	18.62
bSi_2	25.6	493.1	64.6	18.11
bSi_3	20.8	481.9	62.5	13.96
bSi_4	19.2	489.4	60.3	12.58

The *J*–*V* curves for the PV cells fabricated using different samples of planar cSi and nanoporous bSi are shown in Fig. 6. As observed from the Table 5, *η*_avg_ of the planar cSi reference PV cell is 6.93% with *J*_sc_ of 12.5 mA cm^−2^, *V*_oc_ of 453.9 mV and FF of 54.9%. On the other hand, the nanoporous bSi PV cells exhibit *η*_avg_ of 18.62% with *J*_sc_ of 26.8 mA cm^−2^, *V*_oc_ of 510.1 mV and FF of 61.3%. The improved efficiency represents an enhancement of 169% when compared to the planar cSi reference cell. This improvement in the efficiency is contributed by the improved broadband light absorption by the nanoporous bSi. For the nanoporous bSi PV cells, *J*_sc_ exhibits values from 19.2 mA cm^−2^ to 26.8 mA cm^−2^, which is inversely related to the *R*_avg_ of the nanoporous bSi, *i.e.*, *J*_sc_ (26.8 mA cm^−2^) is the highest for the PV cell fabricated on nanoporous bSi with the lowest values of *R*_avg_ (13.7%) and with the smallest average depth (419 nm) of nanopores because the reduced surface area of the shallower nanopores suppresses the surface recombination and responds to blue light more efficiently as compared to the deeper nanopores.^53–55^

The *V*_oc_ for the planar cSi is 453.9 mV while it varies from 481.9 mV to 510.1 mV for the nanoporous bSi PV cells without any special passivating material except SiO_2_ developed during the co-firing of metal contacts at 600 °C for 22 minutes.^42^ The relatively low values of the *V*_oc_ for all the samples is due to the defects introduced during the diffusion of the n-emitter and they act as recombination centers.^54^ The higher values of *V*_oc_ of nanoporous bSi PV cells as compared to planar cSi PV cell could be attributed to the higher bandgap energy (∼1.9 eV) of the nanoporous bSi.^55,56^ Moreover, among the nanoporous bSi PV cells, the *V*_oc_ reduces due to the increased effective surface area of the nanopores on their surfaces which leads to more surface recombination and results in lower values of *V*_oc_.^27,57^ Nevertheless, the nanoporous bSi PV cells demonstrate decent values for *V*_oc_ in absence of any passivating layer. Despite the *J*–*V* characterization under the white light LED source, the maximum *η*_avg_ is 18.62%, which is an improvement over the highest efficiency of 17% measured under AM1.5G illumination (STC) of a PV cell fabricated by using nanoporous bSi synthesised *via* a copper (Cu)-assisted chemical etching reported by Toor *et al.*^27^ This improvement in the efficiency may be attributed to the absence of recombination centres that might have caused by the residues of Al atoms as an impurity during etching process while residues of Cu atoms were left behind in the nanoporous bSi after sonicating the samples in the concentrated solution of HNO_3_ in the work reported by Toor *et al.*^27^ The highest efficiency (18.62%) reported in this work is comparable to the efficiencies of the state-of-art PV cells without any ARC or passivation reported in literature as listed in Table 1.

## Conclusions

In this research, PV cell fabricated using nanoporous bSi synthesized *via* AACE process has been demonstrated for the first time. During the fabrication of the PV cell, diffusion of n-emitter into p-type nanoporous bSi samples increases the average reflection (*R*_avg_) from the surface of the as-synthesized bSi samples. The as-fabricated pn-junction with the lowest value of *R*_avg_ (13.7%) has the surface morphological parameters (*i.e.*, average depth, average diameter, surface coverage and RMS roughness) of 419 nm, 38.7 nm, 42% and 58.9 nm respectively. From the *I*–*V* characterization results of the PV cells, the highest *η*_avg_ obtained from the nanoporous bSi PV cell is 18.62%, which represents an enhancement of 169% over the efficiency of the reference planar cSi PV cell (6.93%) fabricated under the same conditions. The performance improvement of the bSi solar cell is attributed to the improved broadband light absorption (or lower value of *R*_avg_) by the nanoporous bSi material within the 300–1100 nm wavelength region. The efficiency of the solar cell can be further improved by incorporating an ARC and a passivation material on the device.

## Data availability

The authors declare that the raw data supporting this article are available from the corresponding author upon reasonable request.

## Author contributions

All persons who meet authorship criteria are listed as authors, and all authors certify that they have participated sufficiently in the work to take public responsibility for the content, including participation in the concept, design, analysis, writing, or revision of the manuscript. Furthermore, each author certifies that this material or similar material has not been and will not be submitted to or published in any other publication before its appearance in the journal “Silicon”. SU: conceptualization, experimental design, acquisition and analysis of data, manuscript writing, approval of final manuscript for submission, corresponding author. MRH: supervision, resources, reviewing intellectual content of manuscript, approval of final manuscript for submission. MZP: supervision, resources, conceptualization, acquisition and analysis of data, reviewing intellectual content of manuscript, approval of final manuscript for submission.

## Conflicts of interest

The authors declare no competing interests.

## Supplementary Material

RA-015-D5RA01468A-s001
