# Supplementary material for: Photovoltaic cell fabricated using nanoporous black silicon synthesized via aluminium-assisted chemical etching
Source: RSC Adv. 2025 Jun 3;15(23):18566–76. doi: 10.1039/d5ra01468a (PMC12131787; doi:10.1039/d5ra01468a)
Supplement: RA-015-D5RA01468A-s001 [file RA-015-D5RA01468A-s001.pdf]

Supplementary Information (SI) for RSC Advances.
This journal is © The Royal Society of Chemistry 2025

Supplementary Information

Photovoltaic cell fabricated using nanoporous black silicon synthesized via aluminium-assisted chemical etching

Shahnawaz Uddin[1,2], Nur Afidah Md Noor[1,3], Md Roslan Hashim[1], Mohd Zamir Pakhuruddin[1,4*]

[1]School of Physics, Universiti Sains Malaysia, 11800 Minden, Penang, Malaysia.

[2]University Women's Polytechnic, Aligarh Muslim University, Aligarh-202002, India.

[3]JA Solar Malaysia Sdn. Bhd., Lot 17001, Medan Bayan Lepas, Phase 4, Bayan Lepas Industrial Park, 11900 Penang, Malaysia.

[4]Institute of Nano Optoelectronics Research and Technology (INOR), Universiti Sains Malaysia, 11800 USM, Penang, Malaysia.

*Corresponding author: shahnawazuddin.uwp@amu.ac.in

The nanoporous black silicon (bSi) material samples are synthesized via a low-cost aluminium assisted chemical etching (AACE) process as reported in our earlier work [1-4]. The as-synthesized nanoporous black silicon (bSi) samples are characterized and investigated for varying the volume concentration of H_2O_2 in the etching solution of HF-H_2O_2-H_2O (10-x-10 ml) [4]. The effects of varying the chemical composition of the etching solution are observed on the morphological and optical properties of the nanoporous bSi samples [4].

Methodology

Czochralski (Cz) grown, 280 µm thick, p-type monocrystalline silicon (mono cSi) wafers with <100> orientation and resistivity of 1-10 Ω-cm are used in this work. After RCA (RCA-1 and RCA-2) cleaning process, the cSi wafers are rinsed in deionized water (H_2O) and dried by blowing nitrogen (N_2) gas [5]. A thin layer of Al catalyst (24 nm) is deposited on the cleaned cSi samples via direct current (DC) sputtering (model: Auto HHV500 sputter coater). The sputtering uses power of 40 W, base pressure of 4.5×10^{-5} mbar, deposition pressure of 2.7×10^{-3} mbar and Argon (Ar) gas flowing at 10 sccm with average deposition rate of 0.4 nm s^{-1}. Then, the wafers are

annealed at 400 ^{0}C in N_2 atmosphere (flow rate of 2.0 L min^{-1}) inside a furnace (model: LENTON VTF1260700). The purpose of the annealing is to induce dewetting of the Al film, which transforms it into a porous film with irregular and randomly distributed nanoholes [1-4]. After the annealing, wet chemical etching of the samples is carried out in an aqueous solution of an etching agent, HF acid (48-50%) and an oxidizing agent (H_2O_2) (30-32%) by varying the volume concentration of H_2O_2 (1-10 ml) with a constant concentration of HF (10 ml) in 10 ml of H_2O at the room temperature (~25 ^{0}C) for 30 minutes.

The as-fabricated nanoporous b-Si samples undergo the morphological and optical characterizations using field emission scanning electron microscope (FESEM, model: NOVASEM 450), atomic force microscope (AFM, model: Dimension Edge, Bruker), and Agilent's UV-Vis-NIR spectrophotometer (model: Cary 5000) respectively. Hall effect measurements are done using Van der Pauw geometry method at room temperature (25 ^{o}C), probe current of 0.129 mA and magnetic field strength of 0.520 Tesla. During the optical characterization, the total reflection, $R(\lambda)$ (diffused + specular) is measured with an incident angle (8°) of the source light. The ImageJ software is used to analyze the FESEM images for evaluating average depth, average diameter and surface coverage of the nanopores. AFM images are used to calculate the average root mean square (RMS) surface roughness. From the optical results, the weighted average reflections (R_{avg}) of the planar cSi and nanoporous bSi samples are evaluated by using Equation (S-1), within 300-1100 nm wavelength range; where $S(\lambda)$ is photon spectral density (PSD) under AM1.5G (standard solar spectra) [1-4].

$$R_{avg} = \frac{\int_{300\,nm}^{1100\,nm} R(\lambda)\,S(\lambda)\,d\lambda}{\int_{300\,nm}^{1100\,nm} S(\lambda)\,d\lambda} \qquad (S1)$$

Effect of varying the volume concentration H_2O_2 in HF-H_2O_2-H_2O (10-x-10 ml)

As observed in Table S1, there is a corresponding increase in average depth of nanopores on bSi for an increase of concentration levels of H_2O_2 from 1 ml to 7 ml, which is attributed to the higher

generation rate of h^+ resulting in rapid etching of cSi underneath the Al catalyst. When the concentration of H_2O_2 is increased further to 10 ml, the average depth is slightly reduced due to the trimming of the nanopores from their top ends in the lateral direction by HF. On the other hand, the diameter of nanopores continues to increase (from 38.7 nm to 48.9 nm) with the higher concentration levels of H_2O_2 (from 1 ml to10 ml) due to the faster transport of excess holes in the lateral direction as compared to the vertical direction (i.e., to the Al-Si interface). Thus, the average diameter of nanopores shows an upward trend with of the concentration of H_2O_2 in the etching solution. However, the surface coverage reduces with increasing the H_2O_2 concentration due to elimination of shallow nanopores by enhanced lateral etching rate. The surface coverage by the nanopores shows an inverse relationship with their average diameter, i.e., the nanopores with lower diameter are denser than the nanopores with larger diameter.

Table S1. Morphological parameters of planar cSi and nanoporous b-Si fabricated via ACCE process with a varying the volume concentration of H_2O_2 in the etching solution of HF-H_2O_2-H_2O (10-x-10 ml), Al catalyst thickness = 24 nm, annealing temperature = 400 ^oC, etching time = 30 minutes.

**Sample name**	**Volume concentration of H_2O_2 (ml)**	**Average depth of nanopores (nm)**	**Average diameter of nanopores (nm)**	**Surface coverage (%)**	**RMS roughness (nm)**
Planar cSi	NA	NA	NA	NA	1.2
bSi_1	1	436	30.7	45.2	35.7
bSi_2	4	560	35.5	38.5	47.1
bSi_3	7	612	37.8	34.1	52.9
bSi_4	10	556	39.9	30.2	46.7

The broadband light reflection curves (within 300-1100 nm wavelength range) of planar cSi (as a reference) and nanoporous bSi are illustrated in Figure S1 (a). As observed from Table S2, the high average broadband reflection (~40%) in planar cSi is attributed to a step change in refractive index from air to cSi (i.e., for air, $n_{air} = 1$ and for cSi, $n_{cSi} = 4$) while a graded refractive index in an inhomogeneous medium (i.e., nanopores of bSi) causes the incident light trapping resulting in reduction of the light reflection [1-4,6,7]. The same light trapping effect can be observed in Figure

S1 (a-b), where the broadband reflection as well as R_{avg} from the surface of nanoporous bSi is lower as compared to that from planar cSi substrate. The correlation of average depth of nanopores, surface coverage and R_{avg} of the as-fabricated nanoporous bSi samples via ACCE process with a varying volume concentration of H_2O_2 is illustrated in Figure S2 which explains that R_{avg} is minimum (5.7%) when the nanopores are shallower and denser for a low volume concentration of H_2O_2 (1 ml).

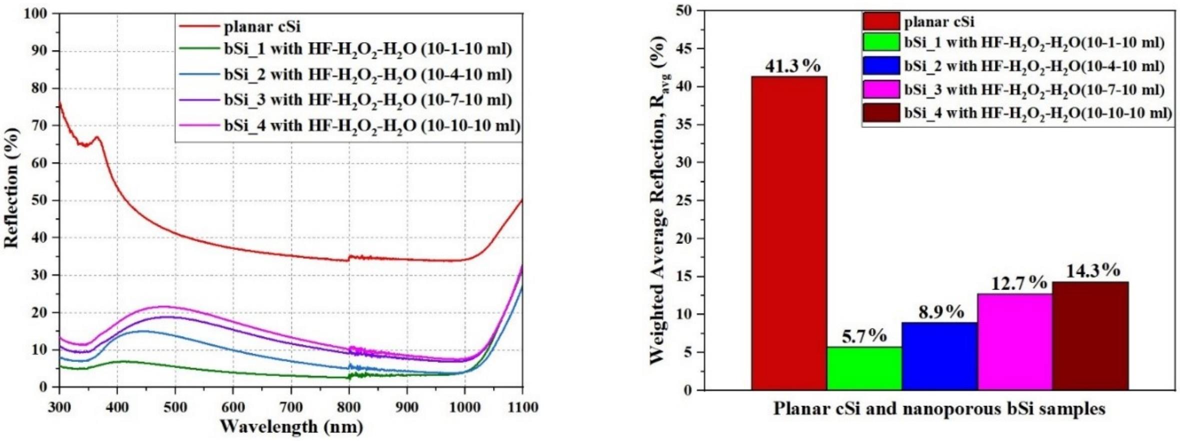

(a)

(b)

Figure S1. (a) Broadband reflection profiles of planar cSi and nanoporous bSi samples (b) R_{avg} of planar cSi and nanoporous bSi samples.

Table S2. R_{avg} of planar cSi and nanoporous bSi samples fabricated by varying the volume concentration of H_2O_2 in the etching solution of HF-H_2O_2-H_2O (10-x-10) ml, Al catalyst thickness = 24 nm, annealing temperature = 400 °C, etching time = 30 minutes.

**Sample name**	**Volume concentration of H_2O_2 (ml)**	**Weighted average reflection (R_{avg}) (%)**
Planar cSi	NA	41.3
bSi_1	1	5.7
bSi_2	4	8.9
bSi_3	7	12.7
bSi_4	10	14.3

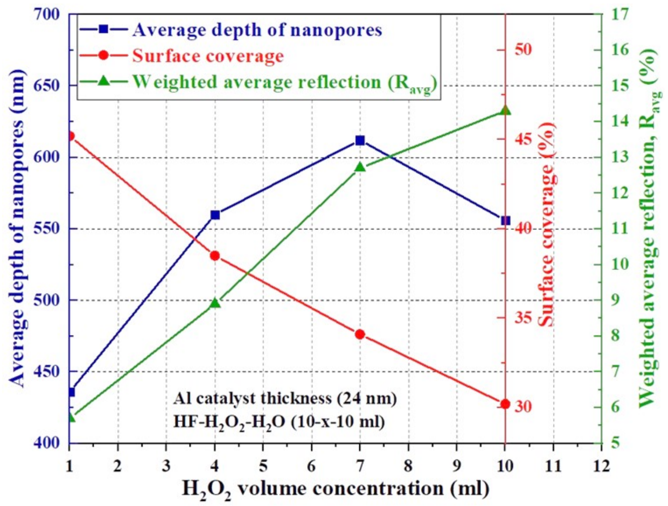

Figure S2. Correlation of the average depth, surface coverage and R_{avg} of nanoporous bSi samples fabricated with a varying chemical composition of $HF-H_2O_2-H_2O$ (10-x-10 ml) by varying the volume concentration of H_2O_2 (1-10 ml).

References

[1] S. Uddin, M.R. Hashim, M.Z. Pakhuruddin, Aluminium-assisted chemical etching for fabrication of black silicon, Mater Chem Phys. 265 (2021). https://doi.org/10.1016/j.matchemphys.2021.124469.

[2] S. Uddin, M.R. Hashim, M.Z. Pakhuruddin, Effects of annealing temperature towards properties of black silicon fabricated by aluminium-assisted chemical etching, Mater Sci Semicond Process. 133 (2021). https://doi.org/10.1016/j.mssp.2021.105932.

[3] S. Uddin, M.R. Hashim, M.Z. Pakhuruddin, Broadband light absorption enhancement in nanoporous black silicon synthesized by aluminium-catalyzed chemical etching, Opt Mater (Amst). 134 (2022) 113111. https://doi.org/10.1016/j.optmat.2022.113111.

[4] S. Uddin, M.R. Hashim, M.Z. Pakhuruddin, Broadband light absorption enhancement in nanoporous black silicon fabricated via aluminium-assisted chemical etching, Silicon (2025). https://doi.org/10.1007/s12633-025-03259-x).

[5] W. Kern, The Evolution of Silicon Wafer Cleaning Technology, J Electrochem Soc. 137 (1990) 1887–1892. https://doi.org/10.1149/1.2086825.

[6] S. Wolin, Ray deflection through a medium having a continuously varying refractive index. JOSA. 43(5) (1953) 373-375. https://doi.org/10.1364/JOSA.43.000373.

[7] S. Chattopadhyay, Y.F. Huang, Y.J. Jen, A. Ganguly, K.H. Chen, L.C. Chen, Anti-reflecting and photonic nanostructures, Materials Science and Engineering R: Reports. 69 (2010) 1–35. https://doi.org/10.1016/j.mser.2010.04.001.
